# Quality of Life, Health and the Great Recession in Spain: Why Older People Matter?

**DOI:** 10.3390/ijerph18042028

**Published:** 2021-02-19

**Authors:** Carla Blázquez-Fernández, David Cantarero-Prieto, Marta Pascual-Sáez

**Affiliations:** 1 Department of Economics, University of Cantabria, 39005 Santander, Spain; david.cantarero@unican.es (D.C.-P.); marta.pascual@unican.es (M.P.-S.); 2 Research Group of Health Economics and Health Services Management—Research Institute Marqués de Valdecilla (IDIVAL), 39011 Santander, Spain

**Keywords:** Spain, quality of life, older people, recession, SHARE, logistic regressions

## Abstract

The financial crisis of 2008 precipitated the “Great Recession”. In this scenario, we took Spain as a country of study, because although it experienced significant negative shocks associated with macroeconomic variables (GDP or unemployment), its welfare indicators have been marked by limited changes. This study used data from waves 2 and 4 (years 2006–2007 and 2010–2012, respectively) of the Survey on Health, Aging and Retirement in Europe (SHARE). Specifically, through logistic regressions we have analysed the effects of socioeconomic, demographic, health and “Great Recession” factors on the quality of life (QoL) of elders in Spain. Although QoL did not change too much during the “Great Recession”, the results confirmed the importance of several factors (such as chronicity) that affect the satisfaction with the QoL among the older people. In this regard, statistically significant effects were obtained for individual exposure to recession. Therefore, a decrease in household income in the crisis period with respect to the pre-crisis period would increase by 44% the probability of reporting a low QoL (OR = 1.44; 95% CI: 1.00–2.07). Furthermore, gender differences were observed. Health and socioeconomic variables are the most significant when determining individual QoL. Therefore, when creating policies, establishing multidisciplinary collaborations is essential.

## 1. Introduction

Adverse trends in economic indicators have occurred across developed countries in the last decade. Specifically, we are referring to the financial crisis of 2008 that precipitated the “Great Recession”. This circumstance has both positive and negative outcomes [1]. However, its long-lasting effects are more remarkable. Explanations are based on the austerity policies introduced in recent years, including cuts to public policies and thus changes to the welfare state benefits [2]. Focusing on ageing people, here we concentrate on the ones associated with quality of life (QoL).

Population aging has been creating new challenges to maintain, and improve, the well-being of individuals in general, and for the elderly in particular. The higher costs that this demographic scenario imposes, because of a higher use of both healthcare services and social resources, could explain that fact. Furthermore, there is uncertainty about the available budgets to be able to cover them [3,4,5].

Therefore, QoL has become one of the key objectives of public policy in modern welfare states. Then, to determine accurate public policies, the main determinants should be recognized. Thus, more additional studies identifying the contributory mechanisms through which changes in both health and socioeconomic factors may affect QoL are needed [6,7,8,9,10,11,12]. In this regard, the primacy of health as the determinant of well-being among the oldest-old is recognized as crucial [8]. Besides, subjective well-being is decisive. Therefore, healthcare systems should be concerned not only with illness and disability, but also with supporting methods to improve psychological states [9]. Overall, the determinants that are correlated with quality of life would include predisposing, health, geographic area and social isolation factors [10].

In addition, although budget cuts due to the “Great Recession” are well known, to what extent the financial crisis of 2008 has contributed to reduced individual well-being is still an open-ended question [13,14].

This study uses data from waves 2 and 4 (pre-recession and recession periods) of the Survey on Health, Aging and Retirement in Europe (SHARE) and logistic regressions to analyse the effects of socioeconomic, demographic, health and “Great Recession” factors on the QoL of elders in Spain. Spain provides a twofold particular scenario. Firstly, Spain together with Japan lead a group of 25 OECD countries with life expectancies over 80 years [15]. Secondly, Spain has been one of the countries most hit by the crisis. In this regard, since 2008, in Spain the number of people at risk of poverty or social exclusion has risen sharply. Besides, it has experienced a great rise in employment rates [16].

In sum, population ageing has created new challenges. This issue is especially significant for countries with aged societies (as in Spain) that have worse health and less socioeconomic resources. The aim of this study is to contribute to the aging literature and to estimate the effect of the latest recession on the quality of life of the oldest people.

The hypotheses here postulated are (a) the QoL of the older people will have on average fallen during the crisis period; (b) reductions in QoL would be directly determined by the “Great Recession” outcomes; and (c) socio-demographic and health variables would also matter for the self-quality of life (subjective well-being). In doing so, our research contributes to both (i) the literature regarding the well-being of the older people and to assess the effect of ageing and health on the life satisfaction of this collective; and (ii) the discussion around the effects of recessions. There is also a question whether the income of an aged population could change during the recession, if most of them are retired; the important of pensions are also behind this issue. Specifically, in this study, it is obtained that a decrease in household income in the crisis period with respect to the pre-crisis period increases by 44% the probability of reporting a low quality of life. Then, multidisciplinary collaborations are highlighted.

This manuscript is structured as follows. The subsequent section describes the data and the econometric model. Afterwards, the empirical findings are presented. The final sections contain the discussion and conclusions.

## 2. Materials and Methods

Three important items in this Section are the data, the empirical strategy and the variables employed in the paper. Previous empirical contributions provide a motivation for the method discussed below.

We explore the effects of the Great Recession on well-being, here measured by QoL, (more precisely, low QoL) by using data from waves 2 and 4 of the Survey on Health, Aging and Retirement in Europe (SHARE). This survey has been specifically established to address multidisciplinary areas of aging. Specifically, we took advantage of easy SHARE, release version: 6.1.1 [17,18,19]. Given that the financial crisis of 2008 precipitated the “Great Recession”, we have used data from 2006–2007 (wave 2) as the pre-recessional period and data from 2010–2012 (wave 4) as the recessional period. In our analyses, we have included Spanish older adults (aged ≥ 50) that answered questions in both waves, *n* = 641.

Our empirical strategy to establish how subjective well-being (*low_QoL*) changed because of the “Great Recession” is based on Boyce et al. [14] and could be synthetized through the following equation:

$$\begin{array}{l} \;\;llow\_QoL_{t}=\beta_{1}low\_QoL_{t-1}+\\ \beta_{2}Individual\;recession\;exposure\;characteristics+\;\beta_{3}Individual\;pre-\\ recession\;exposure\;characteristics\;(Socio\text{-}demographic,\;Health,\;Socialisollation)+\varepsilon . \end{array}$$

That specification includes both the individual recession and pre-recessional characteristics traditionally acknowledged in the empirical literature on health economics. Due to the nature of our dependent variable, we have chosen logistic regression models to estimate the equation/model. Then, we predict *low_QoL* during the recession at *t* (wave 4) from pre-recessional *low_QoL* at *t*−1 (wave 2) such that we estimate the residualised changes in well-being to avoid issues with regression to the mean.

To determine the factors that are the most important contributors to change in well-being over the crisis we have reduced the number of “ideal” variables to simplify the specification and reduce concerns about multi-collinearity. Individual recession exposure characteristics include changes in income, whereas pre-recession characteristics would include socio-demographic (age, gender, education, labour status and geographical location), health and social isolation ones. Furthermore, a correlation matrix was observed. Therefore, the inclusion of all these factors in our model was verified.

In this study, we have articulated individual well-being using a QoL variable, which is measured though variable CASP-12v in the SHARE data. More precisely, this variable is commonly used as measure for well-being as it is both stable between countries and time. CASP-12v ranges between 12 and 48, being interpreted its values as follows: scores QoL below 35 = low QoL; 35–37 = moderate QoL; 37–39 = high QoL; and ≥39 very high QoL. In our sample, the mean is 35.97 ± 5.80. Indeed, here we would focus on *low_QoL*. Our dependent variable is therefore a binary one (1: CASP-12v < 35; 0: otherwise) as we focus only on low quality of life. Regarding the distribution of low QoL across 2006–2007 (pre-crisis) and 2010–2012 (crisis), on average, well-being seems to have shown some worsening. Although the magnitude of the effect is small (2.02%).

Regarding individual recession and pre-recession exposure characteristics, as previously suggested, we have chosen different variables that could better explain the highest reductions in QoL. We have grouped then into recession, socio-demographic, health and social isolation factors. Table 1 shows the variables used, their description and coding.

Specifically, having the household experienced an income decrease from 2006–2007 to 2010–2012 (less_income_r) is considered as a key predictor variable for the recession. As for the socio-demographic factors, we have considered the age of respondent (in 4 intervals), gender (1 if female), educational level (low, middle and high), employment status (employed, retired, unemployed and disabled) and place of residence (rural area or not, 1 if rural). Concerning the health factors: chronic or not (1 if respondent declares any chronic disease). Furthermore, regarding the social isolation factors, we have taken under consideration if the respondent lives alone or not (that is the reason, for example, of not considering here variables related with marital status).

## 3. Results

In this section, we present the empirical results for the model described above based on the recession and pre-recession individual characteristics (Table 3). To fully understand these effects, and as a first approximation to logistic regression results, Table 2 provides the distribution (percentages) for the full sample, disaggregated by gender.

The sample of participants consists of 641 individuals, of which 60.06% are females and have an average age of 65.88 years. It should be highlighted that 41.50% of respondents have experienced reductions in household income through the crisis. Besides, high percentages are observed for rural or chronic variables, with percentages of 51.48% and 64.74%, respectively. Likewise, differences by gender could be also announced. In this regard, a worsening scenario is observed for females, which are somehow younger and with a lower educational level than males. It is also interesting that a higher percentage of men suffer chronic diseases in this sample.

**Figure 1:**
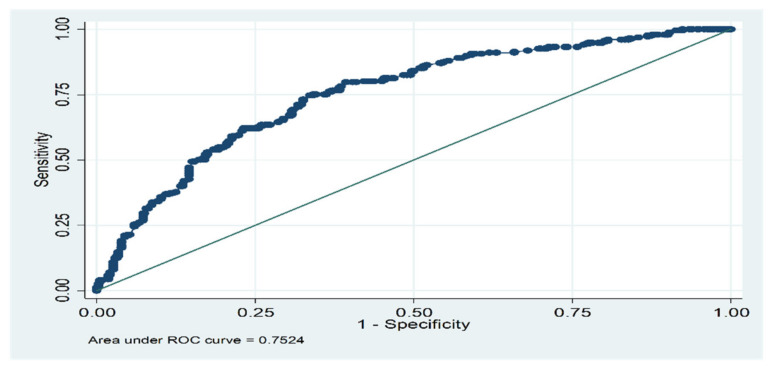
Receiver Operating Characteristic (ROC) analysis. Logistic model for casp_low_r (*n* = 641).

Moreover, Table 3 reports our main estimates for the full sample, and also analyses possible gender divergences. Column 1 presents the OR and Column 2 defines confidence intervals at the 95% level, for each sample. As expected, our empirical findings validate our main hypotheses: low QoL during the crisis period has a large and significant association with both individual recession and pre-recession exposure characteristics. In addition, Figure 1 plots the Receiver Operating Characteristic (ROC) analysis. The relation to the area below the curve is used as a measure of the predictive power of the estimated model. In our approximation, the curve of 0.75 represents acceptable discrimination for the model presented [20].

Therefore, although a low QoL did not change too much during the “Great Recession”, the results confirm the importance of several factors, such as chronicity, that affect the satisfaction with quality of life among the elderly. Statistically significant effects are obtained for individual exposure to recession: an odds ratio of 1.44 means that the odds of a decrease in household income in the crisis period with respect to the pre-crisis period, increases by 44% the probability of reporting a low quality of life. As for the socio-demographic variables, the higher the age and being unemployed or disabled increases low well-being. A reverse effect for those with higher education is shown. However, the rural variables are not significant. No significant effects were found for the social isolation factors. When focusing on gender differences, it is observed that responses in the previous period are more important for males (than for females) whereas age, educational and labour status factors would matter more for females (than for males).

## 4. Discussion

This paper provides new empirical evidence on the factors associated with individual well-being [21]. More precisely, the Spanish aged population was analysed when considering the 2008 economic collapse. Our initial hypothesis was corroborated. Firstly, it has been observed that, although moderate, the QoL of the older people had on average fallen during the crisis period. Secondly, reductions in QoL are directly determined by the “Great Recession” outcomes; and thirdly, both socio-demographic and health variables would also matter for the self-quality of life.

Consequently, our results support previous research indicating that health and socioeconomic variables are the most significant ones when determining well-being, quality of life and/or satisfaction among older Europeans [6,7,10]. Specifically, Angelini et al. [6], for eleven European countries, had highlighted how health problems and physical limitations were potential sources of scale biases for older individuals. Older respondents are more likely to rank themselves as “dissatisfied”. Besides, detrimental health conditions affect the self-assessments (directly and indirectly). In the same line, Stolz [7], for 14 European countries, or Cantarero-Prieto et al. [10], for the southern European ones, reinforced the importance of predisposing, health, geographic area and social isolation factors in explaining the quality of life among the oldest. However, it is obtained that having reported a low QoL in the previous period would be the most important factor determining responses in the following one. Therefore, and anchorage effect is found here. That effect could also explain the fact that place of residence or social isolation factors appear to be not significant. Besides, some differences by gender were observed, and so we had to split the sample in order to test these gender effects. Somehow, differences are observed; so, diverse policies attending to the gender perspective must be considered.

All these issues constitute an essential tool for policymakers when designing policies that target well-being. As a result, the major policy challenge would be to understand those factors determining QoL in order to decrease the number of people at older ages that report a low quality of life.

Moreover, potential limitations and extensions of our study should be mentioned. Despite working with microdata, we should not forget that it is self-reported information, and our recommendations and policy implications should be taken with caution. As for further studies, it should be interesting to explore more variables (such as specific illnesses) and to compare results with other countries in order to gain a better understanding for coordinated social policies.

## 5. Conclusions

The findings of our analysis suggest establishing multidisciplinary collaborations between health professionals, gerontologists, sociologists and economists, in order to understand the complex mechanisms that are connected to quality of life. This cooperation between different social agents would determine the success of public policies in the near future.

## Figures and Tables

**Table 1:** Variables used, and their description and coding.

Variable		Description	Coding
Subjective well-being	Casp_low	Low Quality of life (QoL). The CASP-12v Quality of life and well-being index. Each of its 12 items is answered using a four-point Likert-type scale, and the total score, which ranges between 12 and 48, is interpreted as follows: low QoL, <35; moderate, 35–37; high, 37–39; and very high, ≥39. Our dependent variable is therefore a binary one as we focus only on low quality of life.	1: CASP-12v < 35; 0: otherwise
Recession	Casp_low_r	Low QoL during the crisis	1: if Low QoL during the crisis; 0: otherwise
	less_income_r	Household income changed during the crisis.	1: if household income decreases during the crisis; 0: otherwise
Socio-demographic factors	Age	Age of respondent (four levels in estimates as dummies: 50–59 years, 60–69 years, 70–79 years and ≥80 years)	Years, 1: person is in the age interval; 0: otherwise
	Female	Gender of respondent	1: female; 0: male
	Loweduc	ISCED-97 coding of education, low education	1: low education; 0: otherwise
	Mideduc	ISCED-97 coding of education, middle education	1: middle education; 0: otherwise
	Higheduc	ISCED-97 coding of education, high education	1: high education; 0: otherwise
	Employed	Current job situation	1: respondent is employed; 0: otherwise
	Retired	Current job situation	1: respondent is retired; 0: otherwise
	Unemployed	Current job situation	1: respondent is unemployed; 0: otherwise
	Disabled	Current job situation	1: respondent is permanently sick or disabled; 0: otherwise
	Rural	Area of location (place of residence)	1: respondent lives in a small town, a rural area or village; 0: otherwise
Health factors	Chronic	Chronic diseases	1: respondent reporting any chronic disease; 0: otherwise
Social Isolation factors	Alone	Number of people living in the respondents’ household	1: respondent live alone; 0: otherwise

**Table 2:** Distribution of the analytical sample (%) (*n* = 641; 256 males and 385 females).

Variable		Full Sample	Males	Females
Subjective well-being	Casp_low	38.07	30.07	43.37
Recession	Casp_low_r	40.09	30.47	46.49
	less_income_r	41.50	40.63	42.08
Socio-demographic factors	Female	60.06		
	50–59 years	27.46	23.82	29.87
	60–69 years	37.12	40.63	34.81
	70–79 years	30.11	31.64	29.09
	≥80 years	4.06	3.51	4.42
	Loweduc	86.74	83.98	88.57
	Mideduc	6.55	7.42	5.97
	Higheduc	6.08	8.60	4.42
	Employed	17.00	24.61	11.95
	Retired	37.75	66.41	18.71
	Unemployed	3.28	4.30	2.60
	Disabled	2.65	3.52	2.08
	Rural	51.48	49.22	52.99
Health factors	Chronic	64.74	64.84	64.68
Social isolation factors	Alone	10.60	6.25	13.51

Employment status is determined by four variables (Employed, Retired, Unemployed and Disabled). The shares in these four categories add up to only about 61% for the full sample and 35% for women. They add up to about 99% for the men. The reason is that there appear other categories not included, such as “homemaker” (which takes 62.86% for females). Therefore, when considering Employed (for example), if the woman is “homemaker”, this variable would take a value of 0.

**Table 3:** Logistic regressions models (odds ratios and 95% confidence intervals).

Explanatory Variables			Full Sample (*n* = 641)		Males (*n* = 256)		Females (*n* = 385)	
			OR	95% CI	OR	95% CI	OR	95% CI
Subjective well-being	Casp_low	Yes	3.41	(2.38–4.88) ***	4.85	(2.58–9.11) ***	2.89	(1.86–4.50) ***
		No	1.00		1.00		1.00	
Recession	less_income_r	Yes	1.44	(1.00–2.07) **	1.64	(0.88–3.07)	1.32	(0.84–2.06)
		No	1.00		1.00		1.00	
Socio-demographic factors	Female	Yes	1.97	(1.26–3.09) ***				
		No	1.00					
	Age ^a^	50–59 years	1.00		1.00		1.00	
		60–69 years	1.47	(0.90–2.39)	1.11	(0.40–3.09)	1.62	(0.91–2.87) *
		70–79 years	2.29	(1.33–3.93) ***	1.43	(0.43–4.69)	2.67	(1.41–5.07) ***
		≥80 years	2.47	(0.97–6.26) *	2.95	(0.49–17.79)	2.14	(0.69–6.61)
	Education ^b^	Loweduc	1.00		1.00		1.00	
		Mideduc	0.78	(0.36–1.66)	0.80	(0.23–2.75)	0.72	(0.27–1.91)
		Higheduc	0.33	(0.13–0.87) **	0.60	(0.17–2.04)	0.17	(0.03–0.82) **
	Employment Status ^c^	Employed	1.00		1.00		1.00	
		Retired	1.08	(0.67–1.75)	1.39	(0.49–3.94)	0.97	(0.54–1.74)
		Unemployed	3.76	(1.42–9.98) ***	2.17	(0.46–10.21)	5.93	(1.42–24.71) **
		Disabled	6.98	(2.02–24.09) ***	10.13	(1.58–64.97) **	5.61	(0.87–36.14) *
	Rural	Yes	0.84	(0.59–1.21)	1.13	(0.60–2.15)	0.72	(0.46–1.13)
		No	1.00		1.00		1.00	
Health factors	Chronic	Yes	1.47	(1.00–2.16) **	1.73	(0.88–3.41)	1.27	(0.78–2.06)
		No	1.00		1.00		1.00	
Social isolation factors	Alone	Yes	1.47	(0.83–2.60)	2.20	(0.68–7.10)	1.40	(0.72–2.71)
		No	1.00		1.00		1.00	

***, ** and * indicate significance at the 1%, 5% and 10% level, respectively. Reference category: the reverse one for each dichotomous variable. ^a^ Age is represented through four dummy variables: 50–59 years (reference category), 60–69 years, 70–79 years and ≥80 years. ^b^ Education is categorized in terms of three levels of educational attainment (Loweduc, Mideduc and Higheduc, with Loweduc being the reference category). ^c^ Employment status is determined by four dummies (Employed, Retired, Unemployed and Disabled, Employed being the reference category).

## Data Availability

The data the authors used was obtained from a third party, the SHARE project, and cannot be made available separately by the authors. SHARE data are available to all researchers for purely scientific purposes upon request on their website (http://www.share-project.org/ (accessed on 28 December 2020); contact information: care of Josette Janssen; address: CentERdata, Tilburg University, P.O. Box 90153, 5000 LE Tilburg, The Netherlands; e-mail: jjanssen@uvt.nl; fax: +31 13 4662764).
